# Refining neck dissection strategies in oral squamous cell carcinoma: a surgical perspective

**DOI:** 10.1097/JS9.0000000000002822

**Published:** 2025-07-02

**Authors:** Kai Sun, Keqian Zhi, Ling Gao

**Affiliations:** a Department of Oral and Maxillofacial Reconstruction, The Affiliated Hospital of Qingdao, University, Qingdao, China; b School of Stomatology, Qingdao University, Qingdao, China; c Key Lab of Oral Clinical Medicine, The Affiliated Hospital of Qingdao University, Qingdao, China; d Department of Oral and Maxillofacial Surgery, The Affiliated Hospital of Qingdao University, Qingdao, China

*Dear Editor*,

We commend the authors for the recent review article published in the *International Journal of Surgery* titled *“Robotic-assisted selective and modified radical neck dissection in head and neck cancer patients.”*^[1]^. The article provides a comprehensive discussion on the feasibility, cosmetic outcomes, and intraoperative complications of robotic-assisted neck dissection (RAND), which makes a valuable contribution to improving patient satisfaction in head and neck cancer surgery. As oral and maxillofacial surgeons, particularly in oral squamous cell carcinoma (OSCC), we would like to offer several reflections and practical considerations based on our clinical experience^[2]^.

First, the authors highlighted the significant cosmetic advantages of RAND, particularly among younger female patients. We fully concur with this perspective. Many of my patients also express strong preferences for “hidden incisions.” However, due to the skip-pattern lymphatic metastasis typical of OSCC, level IIb and level Va are common sites of occult nodal metastases^[3]^. In standard open neck dissection, we routinely employ an anterior sternocleidomastoid approach with submandibular triangle dissection and intraoperative nerve monitoring to achieve comprehensive clearance of levels II–V. By contrast, the Robotic-assisted and Modified Facelift Approach (MFL) remains limited in its ability to visualize and dissect level IIb and the space behind the internal jugular vein. Therefore, we suggest that RAND should be reserved for carefully selected low-risk cN0 patients with no radiologic evidence of level IIb/Va involvement. For cN+ patients or those with deep primary lesions (tongue base, oropharynx), open neck dissection should remain the preferred approach to ensure oncologic thoroughness.

Second, for anterior tongue lesions, primary closure often results in satisfactory functional and cosmetic outcomes. However, tumors involving the floor of mouth or ventral tongue often necessitate free flap reconstruction to restore postoperative speech and swallowing. In this context, RAND presents notable limitations. The narrow working channel, rigid instrumentation, limited suturing angles, and lack of tactile feedback greatly increase the technical difficulty and reduce the reliability of microvascular anastomosis. In our practice, we observed a higher incidence of venous congestion, compromised flap perfusion, and even partial flap necrosis when attempting microvascular work under robotic assistance. To address this limitation, we propose a “robotic + open” hybrid strategy—RAND can be used for levels II–IV dissection, while a traditional open window should be maintained for safe microvascular anastomosis during free flap reconstruction. In cases requiring flap reconstruction, conventional open neck dissection should be the primary approach. Cosmetic outcomes can still be optimized through shorter incision lengths and incisions aligned with natural skin tension lines, ensuring that functional reconstruction is not compromised for cosmetic reasons.

Third, for patients with high cosmetic expectations, our center has also refined open surgery techniques, such as adopting submandibular incisions (8–10 cm), designing incisions along relaxed skin tension lines, and using adjunctive therapies such as silicone gel and laser treatments postoperatively. We recommend that scar management protocols be established to address aesthetic concerns in non-robotic cases, to avoid the oversimplification of equating cosmetic outcomes solely with robotic surgery.

It is undeniable that robotic surgery may become an ideal tool for highly selective early-stage N0 patients prioritizing cosmetic outcomes. However, as the review pointed out, much of the current data originate from South Korean cohorts, primarily focusing on papillary thyroid carcinoma. There is a paucity of data for OSCC, and most follow-up periods are less than 2 years. In our center’s retrospective analysis of 349 OSCC patients, we observed that open neck dissection significantly improved disease-free survival^[4]^. Currently, there remains a lack of long-term evidence regarding regional control and recurrence rates following RAND in high-risk OSCC patients.

Therefore, we recommend that future research efforts focus on building dedicated clinical cohorts of OSCC patients undergoing RAND, with a minimum of 3 years of follow-up. Important endpoints should include lymph node ratio (LNR), recurrence patterns, long-term oncologic outcomes, and functional recovery.

In conclusion, while RAND offers distinct cosmetic advantages and represents a valuable option for selected patients, current evidence supports its role as a selective adjunct rather than a routine replacement in the surgical management of OSCC^[5]^. Greater emphasis should be placed on achieving comprehensive lymphatic clearance, long-term oncologic outcomes, and functional preservation in this biologically heterogeneous and high-recurrence cancer type. Future research should go beyond evaluating short-term feasibility and cosmetic outcomes, and instead prioritize robust data on long-term oncologic efficacy and postoperative functional outcomes (such as shoulder function and nerve preservation) in OSCC patients.

## Data Availability

Not applicable.
